# Efficacy analysis of iptacopan in a patient with thrombotic microangiopathy after allogeneic hematopoietic stem cell transplantation: a case report

**DOI:** 10.3389/fimmu.2026.1737424

**Published:** 2026-03-02

**Authors:** Zixuan Zhao, Lin Guo, Jiao Ge, Wei Wu, Yanhua Yue, Yan Qiu, Feng Li, Wenxi Hua, Weiying Gu, Yan Lin

**Affiliations:** Department of Hematology, The Third Affiliated Hospital of Soochow University, Changzhou, China

**Keywords:** allo-HSCT, AML, factor B inhibitor, iptacopan, TA-TMA

## Abstract

To investigate the efficacy of Iptacopan in transplantation-associated thrombotic microangiopathy (TA-TMA) after allogeneic hematopoietic stem cell transplantation (allo-HSCT), we report the case of a 43-year-old male with Acute Myeloid Leukemia, Myelodysplasia-Related (AML-MR, with IDH1 and STAG2 mutations) who developed TA-TMA after allo-HSCT. The patient had an initial partial response to the C5 inhibitor eculizumab, but the disease progressed. Oral Iptacopan (200 mg twice daily) was initiated on Day +36. The patient received a total of six sessions of therapeutic plasma exchange and two concurrent doses of defibrotide during the Iptacopan course. After 30 days of Iptacopan treatment, the patient exhibited a significant hematological response, evidenced by a reduction in LDH (758 U/L to 357 U/L), a rise in platelets (17 to 43×10^9^/L), and a drop in C5b-9 (305.32 to 153.70 ng/mL). This biochemical improvement coincided with key clinical outcomes: resolution of proteinuria and the achievement of sustained red blood cell transfusion independence after day +58 and platelet transfusion independence after day +66, marking a decisive turnaround in his TA-TMA course. Treatment with the novel oral complement inhibitor Iptacopan induced significant hematological and clinical responses in this TA-TMA patient, demonstrating its potential therapeutic efficacy and warranting further clinical investigation.

## Introduction

Transplantation-associated thrombotic microangiopathy (TA-TMA) is a serious complication following hematopoietic stem cell transplantation (HSCT), characterized by microangiopathic hemolytic anemia, thrombocytopenia, microvascular thrombosis, and multi-organ dysfunction (1). A recent retrospective meta-analysis reported a TA-TMA incidence of 12% (95% CI, 9% to 16%) (2). Notably, a prospective study in adults found that the incidence of severe TA-TMA within 100 days post-allo-HSCT was as high as 21.8%, carrying a devastating non-relapse mortality (NRM) of 42% (3).

The pathogenesis of TA-TMA, while not fully elucidated, is widely explained by the “multiple-hit hypothesis”. The first hit involves a patient’s inherent predisposition to complement activation (e.g., genetic variants in complement regulatory genes) or pre-existing endothelial injury. The second hit is endothelial toxicity from the HSCT conditioning regimen (e.g., myeloablative therapy). The third hit comprises post-transplant additional insults, such as medications (e.g., calcineurin inhibitors), infections, and graft-versus-host disease (GVHD) (4).

Among the three complement activation pathways, the alternative pathway (AP) plays a critical role in the pathogenesis of TA-TMA. When the AP is activated, C3 undergoes spontaneous hydrolysis to produce small amounts of C3b, which then binds to factor B (FB). This C3b-FB complex is subsequently cleaved by factor D (FD) into two fragments: Ba and Bb. The Bb fragment remains bound to C3b to form C3bBb, the initial C3 convertase of the AP. This convertase is further stabilized by properdin P to form the stable C3 convertase (C3bBbP), a key mediator that amplifies the complement cascade through continuous C3 cleavage. Factor B serves as a pivotal regulatory protein in this cascade. The plasma level of its cleavage product, Ba, serves as a functional biomarker of the AP activity. Although not directly involved in downstream steps, the Ba level is a recognized indirect indicator of systemic complement activation, especially through the AP (5). In 2021, Okamura et al. reported that elevated Ba levels on the 7th day after transplant were significantly associated with the subsequent development of TA-TMA (6).

The gold standard for diagnosis remains tissue biopsy; however, given the difficulty in performing biopsies in this patient population, contemporary criteria proposed by Jodele et al. integrate clinical and laboratory markers (e.g., microangiopathic hemolysis, thrombocytopenia, new or progressive hypertension, proteinuria, and elevated soluble C5b-9) (7, 8). The Jodele criteria are widely endorsed, including by the Chinese Society of Hematology (9).

We report a case of TA-TMA in a patient who responded favorably to the factor B inhibitor Iptacopan, an agent seldom used in adult TA-TMA cases to date.

## Case presentation

A 43-year-old male with a history of previously untreated myelodysplastic syndrome (MDS) presented with a lung infection on July 10, 2024. Laboratory investigations at admission revealed pancytopenia: white blood cell count (WBC) 0.89×10^9^/L, absolute neutrophil count (ANC) 0.22×10^9^/L, hemoglobin (Hb) 55 g/L, and platelet count (PLT) 47×10^9^/L. Comprehensive bone marrow analysis—integrating morphology, immunophenotyping, cytogenetics, and molecular studies—led to a diagnosis of acute myeloid leukemia (AML-MR, with IDH1 and STAG2 mutations).

Induction therapy with the BCL-2 inhibitor venetoclax plus azacitidine (VA) and retinoic acid was initiated on July 12, followed by a consolidation cycle with the VA regimen on September 26. The patient achieved remission with negative minimal residual disease (MRD). Given the unfavorable prognosis associated with MDS-derived AML, the patient proceeded to allo-HSCT. The conditioning regimen, initiated on December 4, 2024, consisted of low-dose total body irradiation (TBI) 3 Gy on day -7, busulfan from day -6 to -4, cladribine 9.6 mg from day -6 to -2, cytarabine 3.8 g from day -6 to -2, and antithymocyte globulin 175 mg from day -4 to -1. Graft-versus-host disease (GVHD) prophylaxis included cyclosporine A, low-dose methotrexate and mycophenolate mofetil. On December 11, the patient received an infusion of cord blood stem cells (sourced from Shanghai Cord Blood Bank) combined with allogeneic peripheral blood stem cells from a haploidentical cousin donor (B+ to O+, HLA 6/10 match).

Neutrophil engraftment was achieved on day 13 post-transplant. On day 21 post-transplant, the patient developed fatigue, general malaise, and progressively worsening hypertension. Laboratory investigations revealed the following: WBC 14.31×10^9^/L, ANC 11.37×10^9^/L, HGB 66 g/L, PLT 48×10^9^/L, alanine aminotransferase (ALT) 136.8 U/L, aspartate aminotransferase (AST) 113.6 U/L, lactate dehydrogenase (LDH) 743 U/L (ULN 250 U/L), urine albumin 3+ positive, albumin-to-creatinine ratio (ACR) 2+ positive. Cyclosporine was promptly discontinued and substituted with methylprednisolone for GVHD prophylaxis (initiated on day +21). Measures to control hypertension and prevent infection were intensified. Furthermore, the soluble C5b-9 (sC5b-9) level measured on day 26 post-transplant was significantly elevated at 305.32 ng/mL (ULN 244ng/mL). Based on the Jodele criteria, which incorporate elevated LDH, proteinuria (as indicated by elevated ACR >2 mg/mg), progressive hypertension, and a marked elevation in sC5b-9, a diagnosis of TA-TMA was established. Eculizumab (900 mg q3d) was initiated but failed to arrest disease progression, evidenced by rebounding LDH and liver enzymes by day +33. Consequently, oral Iptacopan (200 mg twice daily) was initiated on day +36. To bridge the therapeutic transition, six sessions of therapeutic plasma exchange (TPE) were performed (Days +36, +38, +40, +42, +44 and +46), with Iptacopan administered post-procedure to ensure optimal drug exposure. Two doses of defibrotide were administered on Day +37 and Day +39. The patient achieved red blood cell transfusion independence after day +58 and platelet transfusion independence after day +66.

**Figure 1:**
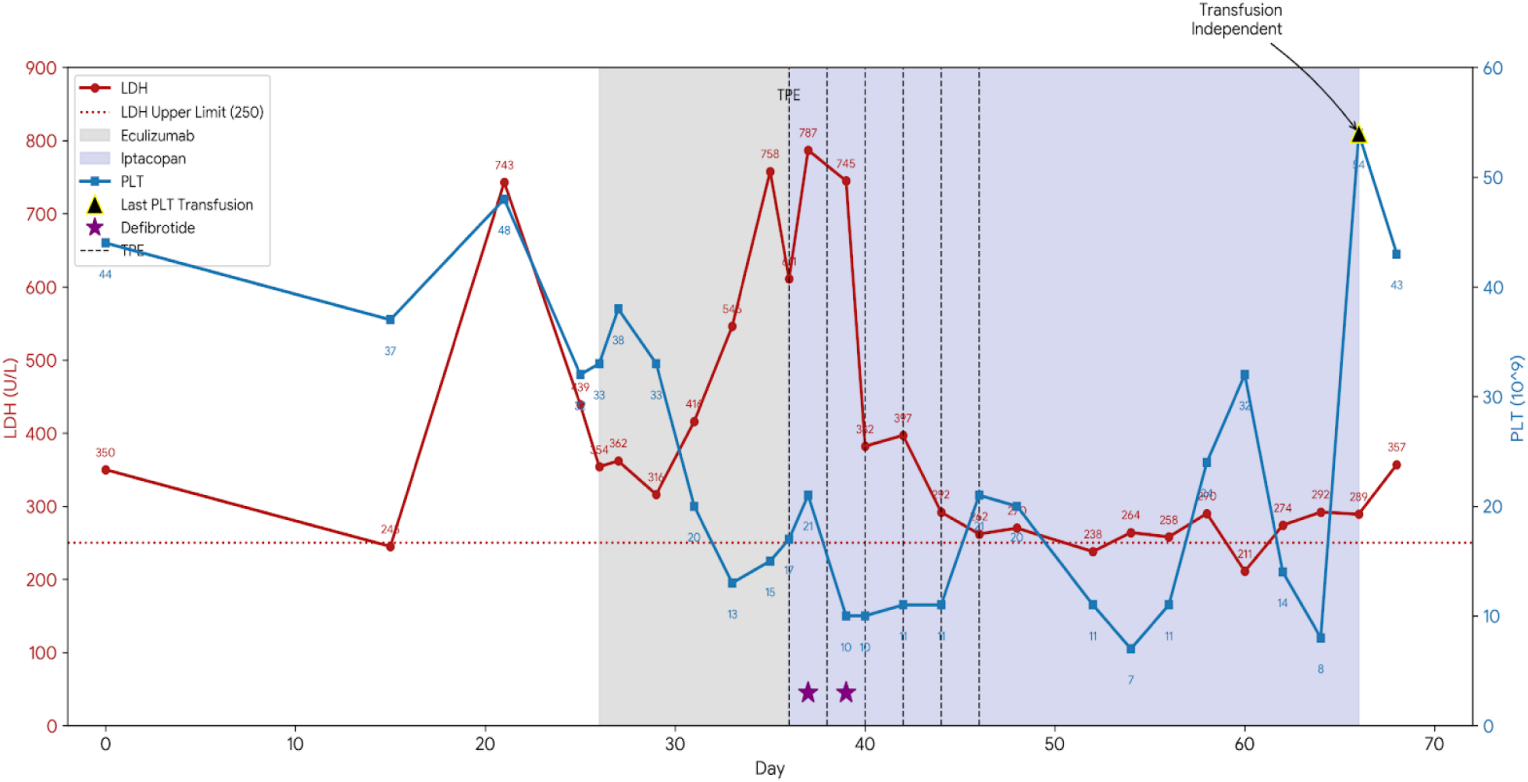
Trends of lactate dehydrogenase (LDH) and Platelet (PLT) counts during the clinical course. The red line represents serum LDH levels (U/L), and the blue line represents platelet counts (10^9/L). The gray shaded area indicates the duration of Eculizumab treatment (Day +26 to +36). The blue shaded area represents the Iptacopan monotherapy phase (Day +36 to +66). Vertical dashed lines denote Therapeutic Plasma Exchange (TPE) sessions. Purple stars indicate the administration of Defibrotide. The black triangle marks the last platelet transfusion, signifying the achievement of transfusion independence (day+66).

**Figure 2:**
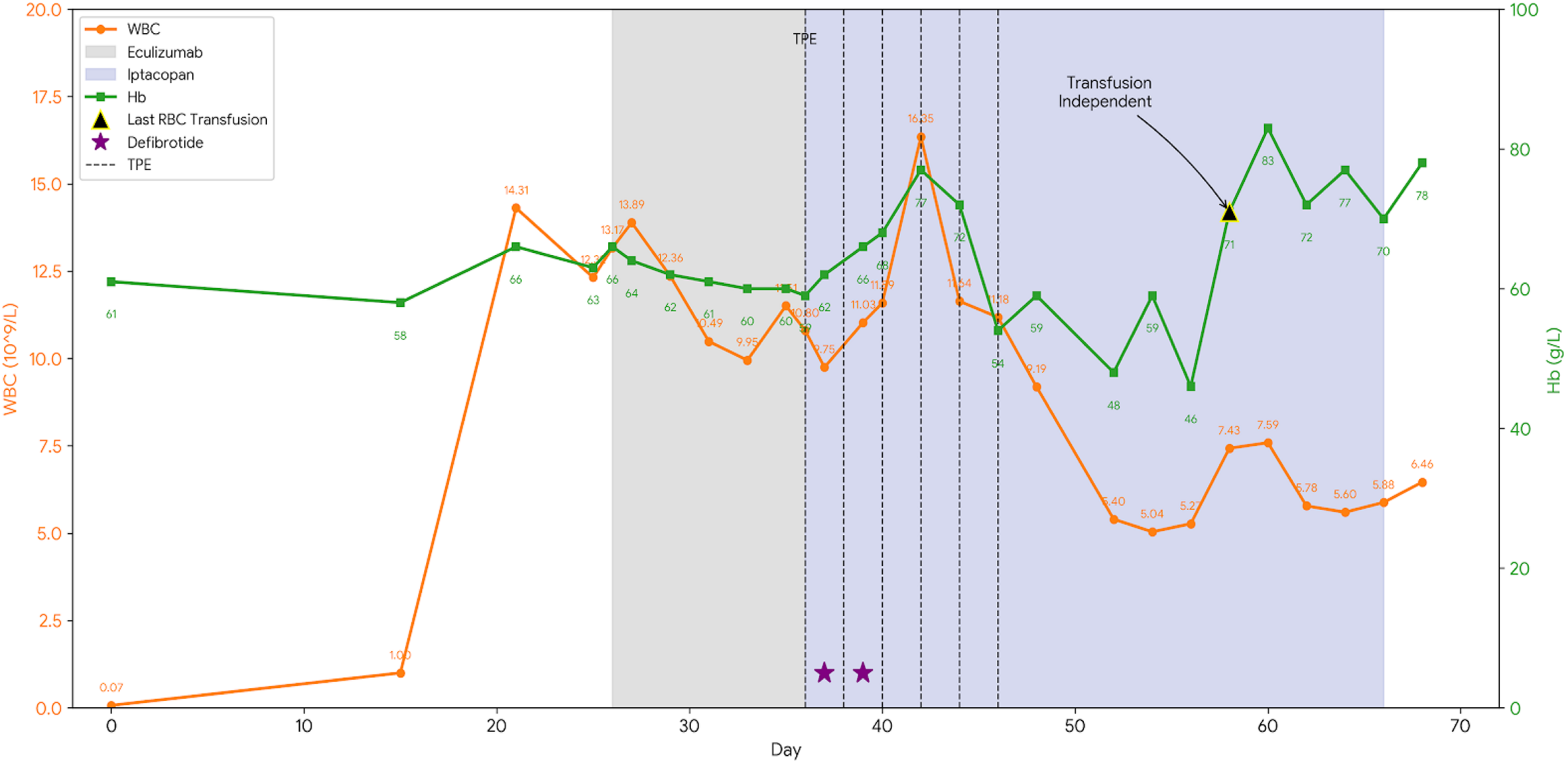
Trends of white blood cell (WBC) and Hemoglobin (Hb) levels. The orange line represents WBC counts (10^9/L), and the green line represents Hemoglobin levels (g/L). Treatment interventions (shaded areas and markers) correspond to those described in **Figure 1**. The black triangle indicates the last packed red blood cell transfusion(day+58).

After 30 days of Iptacopan therapy, the patient showed marked improvement: LDH levels exhibited a pronounced and overall decline, falling from 611 U/L at the start of Iptacopan to 357 U/L. Concomitantly, the platelet count rose from 17 to 43 × 10^9^/L (**Figure 1**). Although initial improvements were noted, transient fluctuations occurred requiring rescue interventions. The final packed red blood cell transfusion was administered on day +58 following a drop in hemoglobin to 46 g/L. Similarly, a platelet dropped to 8×10^9^/L around day +66 necessitated a final rescue platelet transfusion. Sustained transfusion independence for both lineages was achieved after day +66, with subsequent follow-up confirming robust hematopoietic recovery. The leukocyte count, after an initial post-engraftment surge, gradually decreased and stabilized within a normal range (**Figure 2**). This comprehensive biochemical and cellular improvement, achieved within five weeks of Iptacopan therapy, signified a decisive reversal of the thrombotic microangiopathy process. By week 14, the patient exhibited significant clinical improvement. The ACR returned to normal, and the patient was afebrile with normal hepatic and renal function, daily urine output and stool patterns (**Table 1**). Notably, during the course of Iptacopan treatment and follow-up, the patient did not experience any systemic bacterial or fungal infections, including no episodes of neutropenic fever or sepsis. Response to treatment was defined as fulfillment of the following criteria: (1) Normalization of LDH to within institutional upper limit of normal; (2) Achievement of transfusion-independent platelet count >50×10^9^/L; (3) Resolution of proteinuria (negative urine ACR); (4) Control of hypertension without evidence of end-organ damage. The patient met all these criteria by week 14 post-Iptacopan initiation.

**Table 1:** Laboratory parameters at key time points.

Time point (Post-HSCT Day)	Day+21 (Diagnosis)	Day+26 (pre-Eculizumab)	Day+36 (pre-Iptacopan)	Day +44 (1wk post-Iptacopan)	Day +68 (5wks post-Iptacopan)	Day+135 (14wks post-Iptacopan)
sC5b-9 (ng/mL)	305.32	–	–	–	153.70	128.80
LDH (U/L)	743	758	611	292	357	211
Platelet Count (×10^9^/L)	48	15	17	11	43	62
Hemoglobin (g/L)	66	60	59	72	78	83
Proteinuria (ACR)	2+ positive	2+ positive	2+ positive	2+ positive	1+positive	Negative
Hypertension	Yes	Yes	Yes	Improved	Controlled	Controlled

## Discussion

Current Chinese guidelines for the management of TA-TMA recommend both first-line and second-line therapies. First-line treatment primarily involves the withdrawal of causative agents combined with supportive care (4). Key measures include prompt reduction or cessation of calcineurin inhibitors (CNI) or mTOR inhibitors, aggressive hypertension control, and treatment of underlying infections or GVHD (4, 9). The initial management of our patient aligned with established guidelines for TA-TMA. In accordance with this approach, cyclosporine was promptly discontinued upon diagnosis to alleviate the drug-specific endothelial toxicity. This decision was made alongside intensified measures to control hypertension and prevent infection, as recommended by the Chinese consensus (9). We acknowledge that this strategy requires vigilant monitoring for GVHD; however, in this specific case, methylprednisolone was substituted for GVHD prophylaxis. The decision to promptly discontinue CNI is paramount due to its role as a key endothelial trigger in TA-TMA pathogenesis (10). Second-line options include therapeutic plasma exchange (TPE), eculizumab, rituximab, and defibrotide (9). However, clinical experience revealed limitations with these approaches. First-line therapy is often insufficient to halt disease progression, especially in high-risk patients. The efficacy of TPE is variable: it may be effective in patients without gastrointestinal (GI) bleeding but is significantly less effective in those with GI bleeding or active acute GVHD (aGVHD) (11). Although terminal complement inhibitors like eculizumab are in clinical use, they require intravenous administration, are costly, show limited efficacy in some patients (particularly those without dominant classical pathway activation) (12), and carry a risk of serious infections (13). Eculizumab and other C5 inhibitors act at the very end of the complement cascade, preventing the cleavage of C5 into C5a and C5b and thus inhibiting the formation of the membrane attack complex (C5b-9). A key limitation is that this terminal blockade does not inhibit the activity of C3 convertases or the generation of upstream effectors like C3a. This means that endothelial inflammation and injury mediated by components upstream of C5 persist (a phenomenon known as “C3 escape”) (14), potentially leading to incomplete response or relapse. To address these limitations, proximal complement inhibition has emerged as a promising strategy. For instance, inhibiting the lectin pathway with Narsoplimab (a MASP-2 inhibitor) has demonstrated efficacy in TA-TMA by blocking the lectin-driven endothelial injury (15), Although effective, Narsoplimab is not commercially available in China.

Thus, there is an urgent need in clinical practice for novel treatments that are highly effective, orally administrated, precisely target the disease mechanism, and have a better safety profile. Iptacopan, as the first oral drug that specifically blocks complement factor B (FB), meets this unmet clinical need (16). It works by binding directly and specifically to FB, thereby preventing its cleavage by factor D (FD). This inhibition halts the generation of both Bb and Ba fragments. Consequently, the formation and function of the alternative pathway (AP) C3 convertase (C3bBb) are directly blocked, ultimately suppressing AP activation and amplification of complement cascade (17).

Iptacopan has the following significant advantages: 1) Upstream blocking effect. By targeting an upstream component of the complement cascade, it simultaneously inhibits the generation of multiple pathogenic effectors, including C3a, C5a, and the membrane attack complex (C5b-9). This early intervention may thereby more comprehensively prevent complement-caused tissue damage (18). 2)High target specificity. Since abnormal AP activation plays a key role in the pathogenesis of TA-TMA, precisely targeting FB, a central component of the AP, may represent a more effective and specific therapeutic strategy against this disease. 3) Theoretically, by selectively inhibiting the AP, Iptacopan preserves the function of the classical and lectin pathways, which remain capable of combating infectious agents. This proximal inhibition may preserve some immune competence against encapsulated bacteria compared to terminal C5 blockade (19); however, this potential advantage remains hypothetical and requires further clinical validation. Additionally, the oral administration of Iptacopan offers greater convenience in clinic practice and may be better tolerated than intravenous therapies like eculizumab.

Given these theoretical advantages, the favorable outcome observed in our patient provides compelling clinical support for the use of Iptacopan in TA-TMA. However, a critical consideration in this case is the attribution of the sustained clinical response, as the patient received concurrent interventions including therapeutic plasma exchange (TPE) and defibrotide. We acknowledge the concomitant use of these supportive therapies. Crucially, the patient received only two doses of defibrotide (on Day +37 and Day +39), a regimen far below the standard therapeutic course required for sustained endothelial protection and meaningful clinical effect in TA-TMA. Published clinical trials and case series indicate that Defibrotide is typically administered for a minimum of 21 days, or even longer, until clinical resolution is achieved (20, 21). Therefore, the contribution of defibrotide to the observed recovery is likely negligible. While TPE may have provided non-specific support, its effects are known to be transient. The TPE was halted on Day +46, and the sustained and progressive normalization of complement-specific markers (sC5b-9 and LDH) continued well beyond this cessation period. The persistent normalization of complement-specific markers, particularly the significant and sustained reduction in sC5b-9, commenced synchronously with the initiation of Iptacopan and continued well beyond the period of supportive interventions. This temporal association, combined with the mechanistic specificity of sC5b-9 inhibition, strongly suggests that Iptacopan was the primary driver of the therapeutic response.

The rapid decline in LDH and sC5b-9 observed in our case mirrors the complement blockade efficacy reported in the APPLY-PNH trial, where Iptacopan achieved significantly higher rates of hemoglobin stabilization and transfusion independence than anti-C5 agents (22). This mechanistic consistency, combined with the temporal association between drug initiation and biomarker improvement, strongly implicates Iptacopan as the primary driver of TA-TMA resolution. This case demonstrates that Iptacopan can induce a rapid and sustained response in post-transplant TA-TMA. The patient’s fulfillment of key hematological and clinical criteria—including the resolution of microangiopathic hemolysis (normalized LDH), correction of thrombocytopenia (transfusion-independent platelet count >50×10^9^/L), and recovery of end-organ function—aligns with established definitions of TA-TMA response used in contemporary literature (1, 2). Notably, the significant reduction in sC5b-9 provides a mechanism-based explanation for the observed clinical efficacy, underscoring the role of complement dysregulation in this patient’s disease pathogenesis. The timing of the response, which commenced shortly after initiating Iptacopan and was sustained after the cessation of concomitant supportive therapies like plasma exchange, strongly suggests that the proximal complement inhibition was the primary driver of disease remission. This successful therapeutic experience demonstrates the potential efficacy of Iptacopan in treating TA-TMA, particularly for patients’ refractory to or intolerant of conventional therapies. By targeting the alternative pathway, Iptacopan has the potential to overcome the mechanistic limitations of existing C5 inhibitors. Although Iptacopan has shown promise in treating atypical hemolytic uremic syndrome (aHUS) (22) and paroxysmal nocturnal hemoglobinuria (PNH) (23) our case contributes to the rapidly emerging evidence supporting its potential efficacy in TA-TMA (24, 25).

## Limitations

The primary limitation of this report is its single-case design. While the temporal association strongly favors Iptacopan, the initial, intermittent use of both TPE and defibrotide represents confounding factors, precluding a definitive conclusion on single-agent efficacy. Furthermore, long-term follow-up data post-Week 14 (Day +135) are limited to clinical observation rather than a comprehensive laboratory panel, warranting further extended monitoring. Larger-scale prospective studies are warranted to confirm the efficacy and safety profile of Iptacopan in TA-TMA.

## Data Availability

The original contributions presented in the study are included in the article/supplementary material. Further inquiries can be directed to the corresponding author/s.
